# Investigation of Deepfake Voice Detection Using Speech Pause Patterns: Algorithm Development and Validation

**DOI:** 10.2196/56245

**Published:** 2024-03-21

**Authors:** Nikhil Valsan Kulangareth, Jaycee Kaufman, Jessica Oreskovic, Yan Fossat

**Affiliations:** 1 Klick Labs Toronto, ON Canada

**Keywords:** voice, vocal biomarkers, deepfakes, artificial intelligence, vocal, sound, sounds, speech, audio, deepfake, cloning, text to speech, cloned, deep learning, machine learning, model-naive

## Abstract

**Background:**

The digital era has witnessed an escalating dependence on digital platforms for news and information, coupled with the advent of “deepfake” technology. Deepfakes, leveraging deep learning models on extensive data sets of voice recordings and images, pose substantial threats to media authenticity, potentially leading to unethical misuse such as impersonation and the dissemination of false information.

**Objective:**

To counteract this challenge, this study aims to introduce the concept of innate biological processes to discern between authentic human voices and cloned voices. We propose that the presence or absence of certain perceptual features, such as pauses in speech, can effectively distinguish between cloned and authentic audio.

**Methods:**

A total of 49 adult participants representing diverse ethnic backgrounds and accents were recruited. Each participant contributed voice samples for the training of up to 3 distinct voice cloning text-to-speech models and 3 control paragraphs. Subsequently, the cloning models generated synthetic versions of the control paragraphs, resulting in a data set consisting of up to 9 cloned audio samples and 3 control samples per participant. We analyzed the speech pauses caused by biological actions such as respiration, swallowing, and cognitive processes. Five audio features corresponding to speech pause profiles were calculated. Differences between authentic and cloned audio for these features were assessed, and 5 classical machine learning algorithms were implemented using these features to create a prediction model. The generalization capability of the optimal model was evaluated through testing on unseen data, incorporating a model-naive generator, a model-naive paragraph, and model-naive participants.

**Results:**

Cloned audio exhibited significantly increased time between pauses (*P*<.001), decreased variation in speech segment length (*P*=.003), increased overall proportion of time speaking (*P*=.04), and decreased rates of micro- and macropauses in speech (both *P*=.01). Five machine learning models were implemented using these features, with the AdaBoost model demonstrating the highest performance, achieving a 5-fold cross-validation balanced accuracy of 0.81 (SD 0.05). Other models included support vector machine (balanced accuracy 0.79, SD 0.03), random forest (balanced accuracy 0.78, SD 0.04), logistic regression, and decision tree (balanced accuracies 0.76, SD 0.10 and 0.72, SD 0.06). When evaluating the optimal AdaBoost model, it achieved an overall test accuracy of 0.79 when predicting unseen data.

**Conclusions:**

The incorporation of perceptual, biological features into machine learning models demonstrates promising results in distinguishing between authentic human voices and cloned audio.

## Introduction

An increasing number of individuals rely on digital platforms as their primary sources of news and information [1]. People often trust what they consume on the internet without doing any research on the source. There is a technological advancement significantly influencing the production of digital media known as “deepfake.” Deepfake constitutes a synthetic reproduction of media content, both auditory and visual, carefully crafted to closely represent the physical attributes and vocal characteristics of a specific individual. Its use spans many domains, notably in entertainment, where it can be used for the digital replication of actors for special effects or the creation of intricately detailed characters in video games [2].

Deepfakes are generated through the aggregation of substantial data sets, including voice recordings, images, and video segments [3]. This research specifically targets the detection of audio deepfakes, relying solely on voice data for both deepfake development and detection method testing. The voice data sets serve as the foundation for training deep learning models, predominantly deep neural networks, with the primary objective of encoding unique and distinguishable attributes and characteristics found in human voices, like speech patterns and intonation [3]. Following successful model training, it gains the capability to produce replicated voice data by processing input audio or text [3]. While initially trained with substantial data sets, deepfake generation models posttraining can produce new voice clones with minimal audio input, synthesizing voice data to replicate the target voice’s distinctive traits based on learned patterns during the training phase.

This technology is valuable in many domains including voice assistants, voice dubbing for multimedia, professional voiceovers, and the narration of audiobooks [4]. Deepfake content can be generated rapidly once a model is trained, thereby significantly improving efficiency across many industries. Unfortunately, the irresponsible and unethical misuse of deepfakes is prevalent, encompassing impersonation, the dissemination of false information, and violation of privacy [5,6]. Due to the dynamic and rapidly evolving nature of this technology, remaining updated with the ongoing advancements in deepfake detection is challenging [7].

Individuals need a reliable tool to verify that the information they are consuming is authentic. Several outdated deepfake detection machine learning methods have high levels of accuracy, achieving up to 100% accuracy on a data set [8]. However, these accurate predictions are restricted to the level of advancement of the deepfakes that the detection models are trained with [9]. For example, the previously mentioned tool that achieved 100% accuracy was trained and tested on a data set of deepfakes generated in 2019, which are of much lower quality than the level of deepfakes available in 2023 [8]. Furthermore, recent work has shown that out-of-domain voice clone detectors (ie, voice detectors applied outside of the data set in which they were applied) had extremely low performance, obtaining an area under the receiver operator curve (AUC) of 25% [10]. A more robust detection method might involve searching for the absence of biological features in the cloned voice, rather than the presence of digital features [11].

Activities such as respiration, swallowing, and cognitive processes can influence speech production and the pattern of pauses in authentic speech. Although voice cloning processes may closely mimic human speech production, machines have no requirements for speech breaks and instead rely on training data to indicate where these pauses occur. This may result in subtle but detectable differences in the way pauses are present in authentic versus cloned audio. Indeed, when humans were asked to distinguish between audio deepfakes and authentic voices, one of the primary justifications for a fake audio classification was unnatural pauses in the recordings [10]. Furthermore, when these features were integrated into a classification regime, a moderate accuracy (approximately 85%) was achieved when analyzing deepfakes by perceptual features such as the amplitude of speech and pauses within a recording [12]. However, that study only assessed the use of a single voice cloning software (ElevenLabs) and a small number of cloned voices (9 built-in text-to-speech (TTS) voices and voices cloned from 2 celebrities). Furthermore, the training, validation, and testing sets were not split by participants, so it is assumed that recordings from the same participant are present in both the training and testing data sets.

We posit that the absence of regular human vocal biomarkers, characterized by the pause pattern in a speech segment, will be effective in differentiating cloned audio from authentic audio. For a more comprehensive understanding of model performance on out-of-domain data, we test the proposed methodology in the following ways:

1. On real and cloned audio recordings the model was not exposed to during training, including built-in TTS obtained from the cloning models
2. On a paragraph the model was not exposed to during training
3. On a new cloning software the model was not exposed to during training

## Methods

### Recruitment

A total of 49 adult participants (20 male) were recruited for this study between June and August 2023 in Toronto, Canada. The participant pool exhibited diversity in terms of ethnicity and had various types and strengths of accents. Exclusion criteria for recruitment included: (1) any person not living in Canada, (2) any person below the age of 18 years, and (3) any speech pathology or condition impeding the production of standard speech, such as stuttering, vocal cord pathology, tracheostomy, or the common cold. No restrictions on gender, ethnicity, accents, or other demographic data were implemented in the recruitment procedure.

**Figure 1:**
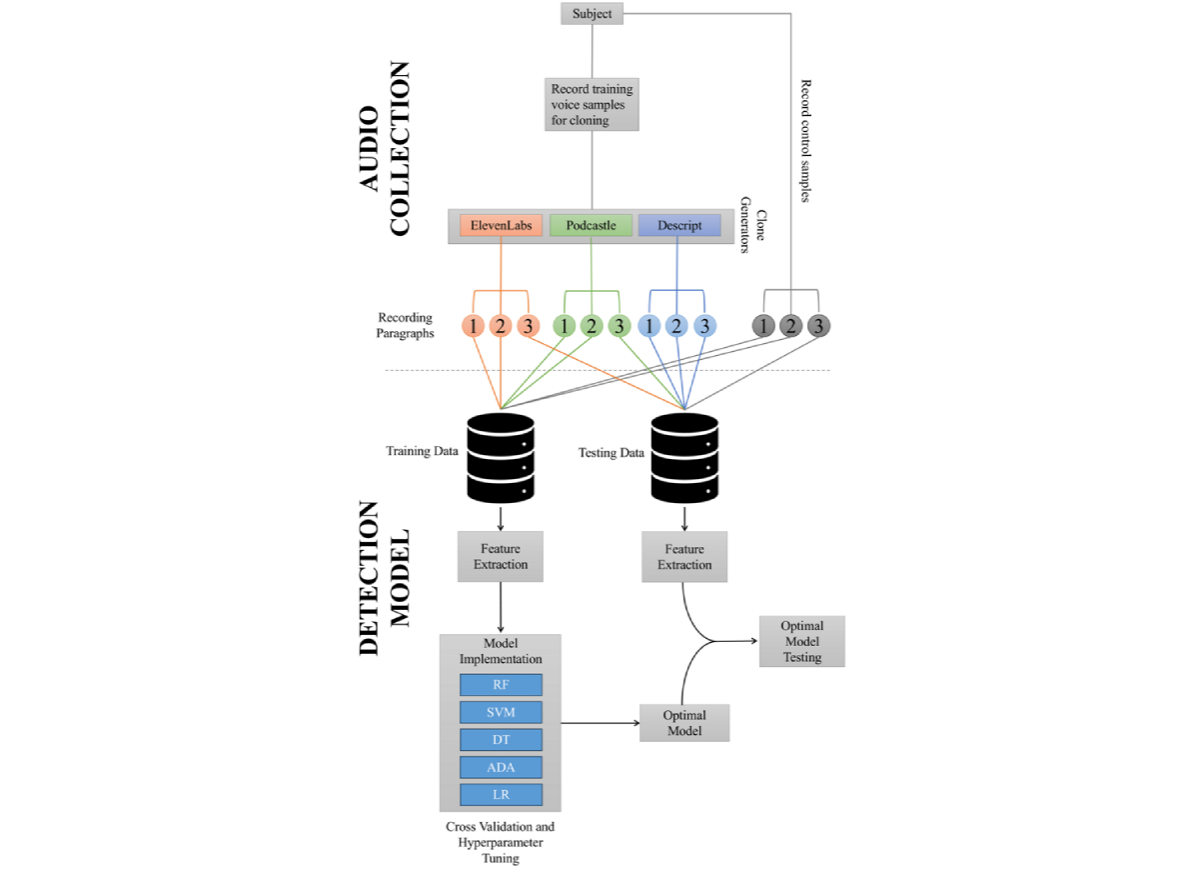
General study protocol overview comprising the audio collection section and detection model development for a participant used in model training. Note that for participants not used in model training (“Model-Naïve Participants”), all data are used for model testing. ADA: AdaBoost; DT: decision tree; LR: logistic regression; RF: random forest; SVM: support vector machine.

The summarized protocol, as illustrated in Figure 1, involves participants recording the required voice samples for the training of 3 distinct deepfake models and a control version of 3 test paragraphs. Subsequently, each deepfake model generates each test paragraph, resulting in a total of 9 deepfake audio samples, in addition to the 3 control samples for each participant. It is worth noting that some participants were unable to complete the necessary training voice recordings for 1 or 2 of the deepfake generators due to time constraints, resulting in varying numbers of recordings and deepfakes among participants.

### Ethics Approval

The research protocol received approval from the Canadian SHIELD Ethics Review Board (REB Tracking Number 2023-06-003).

### Audio Samples

In this study, we generated deepfakes using 3 publicly available and user-friendly web-based models: ElevenLabs [13], Podcastle [14], and Descript [15]. Each of these models required different training data. ElevenLabs had the least specific training requirements and was provided approximately 10 minutes of voice recordings, Descript required 10 minutes of speech samples, and Podcastle required participants to read 70 short phrases.

Recordings took place in a quiet room with participants seated in front of a MacBook Pro with 2.8 GHz Quad-Core Intel Core i7. They were instructed to articulate their speech clearly at a standard speaking volume, using the laptop’s built-in microphone to record. The laptop screen displayed the text that participants were required to read for the collection of voice sample data, including the 3 test paragraphs used in the development of the classification model.

All audio samples were saved in the Waveform Audio Format. The respective voice sample data were input for each deepfake generation model for the training process. Upon completion of the model training, a TTS technique was used to generate deepfake versions of the 3 test paragraphs for each model.

Each voice cloning platform also provides pregenerated TTS voices. We generated each of the 3 paragraphs using all available pregenerated TTS to be used in model testing.

### Feature Generation

**Figure 2:**
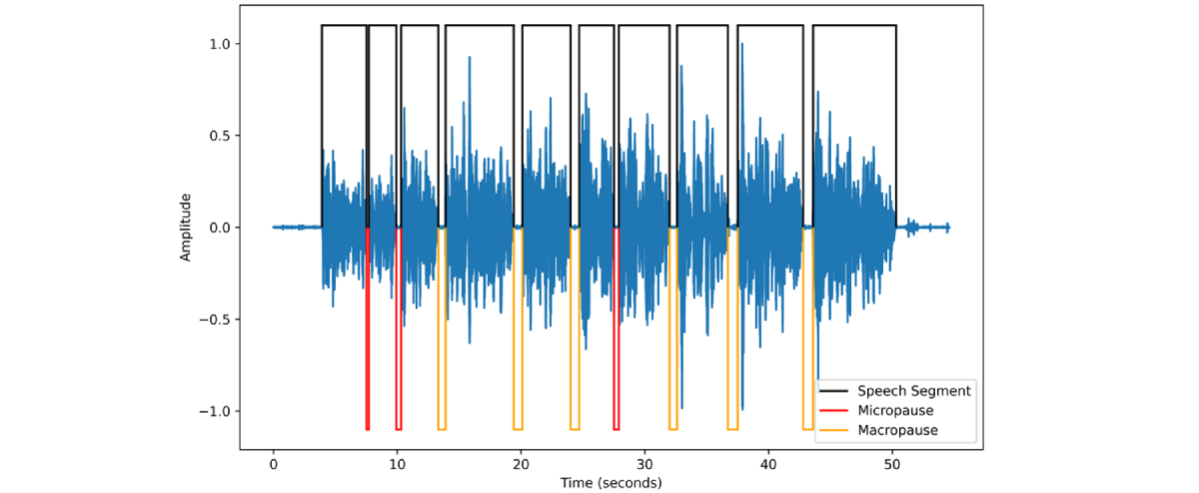
Sample speech and pause illustration. Black segments indicate speech segments, red segments illustrate micro pauses (pauses<0.5 seconds and ≥0.1 seconds), and yellow segments indicate macro pauses (pauses≥0.5 seconds).

The aim of the analysis was to characterize cloned voices using amplitude-agnostic perceptual voice features, primarily characterized by the pause patterns within a speech segment. Speech segments were identified using a voice activity detector (VAD Solero) in Python [16]. The time between speech segments was calculated and classified as a micropause if the time between segments was greater than or equal to 0.1 seconds and less than 0.5 seconds. It was classified as a macropause if the time between segments was greater than or equal to 0.5 seconds (Figure 2). The recording was trimmed so that the recording began at the beginning of the first speech segment and concluded at the end of the final speech segment. Overall, five features were obtained to denote the pause pattern:

1. SpeechAV: The average speech segment length.
2. SpeechSD: The SD of the speech segment lengths.
3. SpeechProp: The proportion of time speaking, calculated by the sum of all the speech segment lengths divided by the length of the entire recording.
4. MiRate: The rate of micropauses, calculated by dividing the number of micropauses by the length of the trimmed recording (in minutes).
5. MaRate: The rate of macropauses, calculated by dividing the number of macropauses by the length of the trimmed recording (in minutes).

Previous work published by Barrington et al [12] evaluated perceptual features to compare audio deepfakes and authentic voices. In this work, 4 summary metrics to characterize the pauses were generated: the average length of a pause, the SD of the pauses, the pause ratio, and the total number of pauses. We slightly modified and expanded these features to align with our hypothesis. Rather than the average length and SD of the pauses, we used the average length and SD of the speech segments. We hypothesized that cloned audio would have longer periods between pauses, as they would have no requirements for biological processes such as breathing or swallowing. Furthermore, instead of reporting the number of pauses, which is dependent on the text spoken and the length of the recording, we exclusively reported pause rates. To account for the differences in pause lengths, we calculated the rates of both micropauses and macropauses.

Contrary to the work published by Barrington et al [12], we chose not to include amplitude features. The amplitude of a voice recording can be influenced by the type of microphone used in recording and the distance of the participant to the microphone. Due to this variation, and the desire to evaluate pause metrics exclusively, we chose to remove amplitude-associated features from our feature set.

### Audio Feature Information

Audio features were compared between authentic and cloned audio. All analysis was conducted in Python. Statistical analysis was conducted using the scipy Python package [17]. *P* values were calculated using the Mann–Whitney U test. Statistical significance is defined as *P*<.05.

### Detection Model Generation

An experiment was conducted to assess 5 models to determine the most suitable machine learning tool for this application: random forest (RF), decision tree (DT), logistic regression (LR), support vector machine (SVM), and AdaBoost (ADA) models. Neural networks, although useful in previous deepfake detection methods, perform best with large amounts of training data and tend to overfit with smaller data sets. We aimed to show speech pause patterns could be used to create a robust model even with a small amount of training data, so neural networks were not included in the current analysis.

A 5-fold stratified group cross-validation was used during model training and hyperparameter tuning to find the optimal model. Paragraphs 1 and 2 in Multimedia Appendix 1, and ElevenLabs and Podcastle generators were used in model training. A total of 30 participants were used in cross-validation (approximately 60% of participants). All recordings corresponding to a participant were kept in the same group, such that if a participant was in one of the folds, all the authentic and cloned recordings obtained from that participant were in the same fold. The total number of recordings used in cross-validation model training is displayed in Table 1.

All analysis was conducted in Python. Models were trained using the scikit-learn Python package [18]. Hyperparameters were tuned using the GridSearch algorithm in scikit-learn*,* using the parameters denoted in Multimedia Appendix 2. Accuracy is defined as



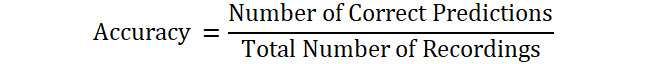



Model performance was assessed by the average balanced accuracy of all folds for a model, defined as



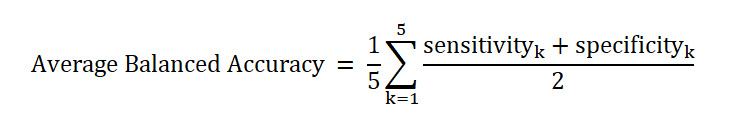



where k is the fold number, sensitivity is the accuracy of the model in predicting audio deepfakes, and specificity is the accuracy of the model in predicting authentic audio.

**Table 1:** Number of recordings collected and generated.

		Training data set (P1^a^/P2^b^), n	Testing data set (P1/P2/P3^c^), n	Total data (P1/P2/P3), n
All recordings		127 (63/64)	257 (63/58/136)	384 (126/122/136)
**ElevenLabs**				
	Pretrained recordings	—^d^	19 (7/5/7)	19 (7/5/7)
	Cloned recordings	45 (22/23)	28 (4/0/24)	73 (26/23/24)
	Total recordings	45 (22/23)	47 (11/5/31)	92 (33/28/31)
**Podcastle**				
	Pretrained recordings	—	53 (18/18/17)	53 (18/18/17)
	Cloned recordings	27 (13/14)	30 (6/4/20)	57 (19/18/20)
	Total recordings	27 (13/14)	83 (24/22/37)	110 (37/36/37)
**Descript**				
	Pretrained recordings	—	6 (2/2/2)	6 (2/2/2)
	Cloned recordings	—	46 (13/16/17)	46 (13/16/17)
	Total recordings	—	52 (15/17/18)	52 (15/17/18)
**Authentic**				
	Total recordings	55 (28/27)	75 (13/13/49)	130 (41/40/49)

^a^P1: paragraph 1.

^b^P2: paragraph 2.

^c^P3: paragraph 3.

^d^Not applicable.

### Optimal Model Testing

The optimal model from the detection model generation was tested on unseen data. For testing, there were three subgroups of data:

1. Audio recordings from individuals the model was not exposed to during training. This subgroup consists of:
  Participant audio recordings that were not used in model training (“Model-Naïve Participants”). Note that for a participant to be “Model-Naïve”, neither authentic nor cloned audio obtained from that participant was used in model training.
  Built-in, pretrained TTS obtained from the cloning models (“Pre-Generated TTS”)
2. A paragraph the model was not exposed to during training (“Model-Naïve Paragraph”; P3, Multimedia Appendix 1).
3. A new cloning software the model was not exposed to during training (“Model-Naïve Generator”). This was the Descript generator.

The model was tested in such a way that each testing datapoint was Model-Naïve in at least 1 of the 3 above subgroups. Data classes used in model training are denoted as “Model-Trained”.

## Results

### Audio Feature Information

The 5 audio features corresponding to the speech pause profiles were calculated from the training data and are displayed in Table 2. Overall, cloned audio was significantly associated with increased time between pauses (*P*<.001), decreased variation in the length of speech segments (*P*=.003), increased overall proportion of time speaking (*P*=.04), and a decreased rate of micro- and macropauses in speech (both *P*=.01).

**Table 2:** Participant and recording data for model features for training data.

Feature	Authentic audio, mean (SD)	Cloned audio, mean (SD)	*P* values^a^
SpeechAV	2.93 (1.76)	3.49 (1.23)	<.001
SpeechSD	1.51 (1.83)	1.22 (0.89)	.003
SpeechProp	0.87 (0.04)	0.89 (0.04)	.04
MiRate	11.72 (4.34)	9.47 (4.25)	.01
MaRate	7.04 (3.39)	5.78 (2.74)	.01

^a^*P* value calculated using Mann-Whitney U test. Statistical significance defined as *P*<.05.

### Detection Model Generation

**Figure 3:**
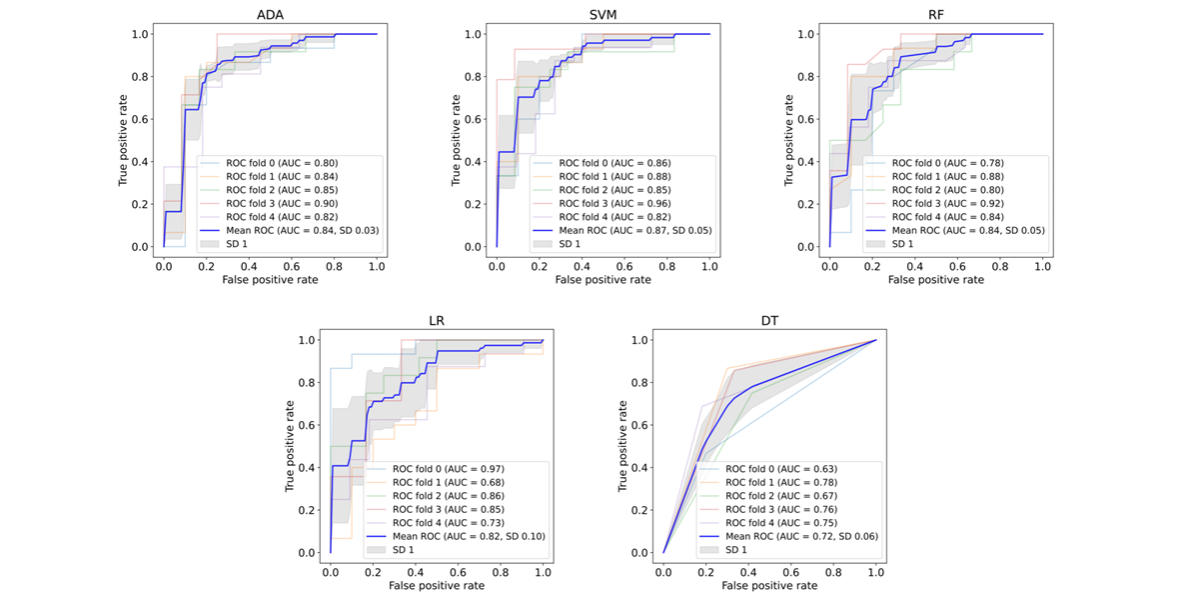
Average receiver operator curves with variability of all models. The results presented are calculated using the optimal parameter set for each model after Grid Search cross-validation. ADA: AdaBoost; AUC: area under the receiver operator curve; DT: decision tree; LR: logistic regression; RF: random forest; ROC: receiver operator curves; SVM: support vector machine.

Five classical machine learning algorithms were implemented to create the prediction model, using the 5 features presented in Table 2. A total of 127 recordings were used to train each model and 257 recordings were used to test each model (see Table 1). The optimal performance was obtained by an ADA model, achieving a 5-fold cross-validation balanced accuracy of 0.81 (SD 0.05). The subsequent models were SVM (balanced accuracy 0.79, SD 0.03) and RF (balanced accuracy 0.78, SD 0.04), followed by LR and DT (balanced accuracies 0.76, SD 0.10 and 0.72, SD 0.06). Unsurprisingly, the models that are traditionally less prone to overfitting (ADA and SVM) were the models that had the best performance, whereas the model that was more likely to overfit (DT) had the poorest performance. Furthermore, ADA and other boosted models can experience the curse of dimensionality when data have many features. By using a small feature set (5 features), we avoided this problem, and ADA achieved a high cross-validated accuracy. Receiver operator curves of all models are shown in Figure 3, and additional model metrics are presented in Table 3. Tuned model hyperparameters are presented in Multimedia Appendix 2.

**Table 3:** Model prediction results for all models.

Model^a^	Balanced accuracy, mean (SD)	Authentic voice accuracy, mean (SD)	Cloned voice accuracy, mean (SD)	Precision, mean (SD)	f1-score, mean (SD)
AdaBoost^b^	0.81 (0.05)	0.75 (0.09)	0.87 (0.08)	0.82 (0.07)	0.84 (0.04)
Support vector machine	0.79 (0.03)	0.73 (0.06)	0.85 (0.05)	0.80 (0.03)	0.82 (0.02)
Random Forest	0.78 (0.04)	0.73 (0.08)	0.83 (0.07)	0.80 (0.07)	0.81 (0.05)
Logistic Regression	0.76 (0.10)	0.70 (0.16)	0.83 (0.09)	0.79 (0.11)	0.81 (0.08)
Decision Tree	0.72 (0.06)	0.71 (0.08)	0.73 (0.15)	0.77 (0.07)	0.73 (0.09)

^a^Results presented are calculated using the optimal parameter set for each model after Grid Search cross-validation.

^b^Optimal model.

### Optimal Model Testing

The optimal ADA model was tested on trained and naïve generators and participants with the paragraphs used in model training (Table 4), and a Model-Naïve paragraph (Table 5). The optimal overall testing performance was obtained when the model was tested on pretrained paragraphs for naïve participants (0.89 overall accuracy). The poorest authentic classification accuracy was obtained when trained participants spoke a new paragraph (accuracy 0.70), potentially indicating the model was overfit to the paragraphs used in training by trained participants. The highest authentic classification accuracy was obtained by model-naive participants speaking model-trained paragraphs with an accuracy of 0.96. Conversely, the detection of cloned and pregenerated voices typically performed better on Model-Naïve paragraphs (most accuracies >0.70). The exception to this was the Model-Naïve Generator which had an overall accuracy of 0.67. However, the number of datapoints for this category was extremely small (N=3) so this accuracy may not be the best representation of the Model-Naïve Generator performance. Pregenerated voices with the trained paragraphs had the lowest performance of all the model testing (overall 0.67 accuracy), but classification performance was much higher in the model-naive paragraph (overall accuracy 0.89). When the results of all confusion matrices in Tables 4 and 5 are compiled, the overall accuracy of all testing data was 0.79 with an AUC of 0.88.

**Table 4:** Confusion matrices of model test results for model-trained paragraphs (P1 and P2).

		Predicted authentic	Predicted fake	Accuracy
**Model-trained participants**				
	Authentic	—^a^	—	—
	Model-trained generator	—	—	—
	Model-naïve generator	5	17	0.773
	Overall	—	—	0.773
**Model-naïve participants**				
	Authentic	25	1	0.962
	Model-trained generator	3	10	0.769
	Model-naïve generator	1	7	0.875
	Overall	—	—	0.894
**Pregenerated TTS^b^**				
	Authentic	—	—	—
	Model-trained generator	17	31	0.646
	Model-naïve generator	0	4	1.00
	Overall	—	—	0.673

^a^Not applicable.

^b^TTS: text-to-speech.

**Table 5:** Confusion matrices of model test results for the Model-Naïve paragraph (P3).

		Predicted authentic	Predicted fake	Accuracy
**Model-trained participants**				
	Authentic	19	8	0.704
	Model-trained generator	7	29	0.806
	Model-naïve generator	0	14	1.00
	Overall	—^a^	—	0.805
**Model-naïve participants**				
	Authentic	16	6	0.727
	Model-trained generator	1	7	0.875
	Model-naïve generator	1	2	0.667
	Overall	—	—	0.758
**Pregenerated TTS^b^**				
	Authentic	—	—	—
	Model-trained generator	3	21	0.875
	Model-naïve generator	0	2	1.00
	Overall	—	—	0.885

^a^Not applicable.

^b^TTS: text-to-speech.

## Discussion

### Principal Findings

This paper outlines the development of an audio deepfake detection model that capitalizes on the distinctive biological vocal characteristics to distinguish between genuine human speech and machine-generated audio. Voice clone samples were created for each participant using 3 publicly available platforms: Descript, ElevenLabs, and Podcastle. To compare these cloned samples with the participants’ authentic voice recordings, a variety of perceptual features were calculated to characterize the pause pattern in a recording. The hypothesis was that the speech and pause pattern would be distinguishable between authentic voice recordings and voice clones, as a machine-generated audio sample would not be under the same biological requirements as a human. Machines have no requirements for breathing or swallowing, and their processing time is magnitudes shorter than humans. Even if machines falsely replicate the pauses in speech, their lack of necessity for these processes may create subtle distinctions in the overall pause patterns. Our results support this finding, and 5 perceptual pause features were used to create a detection model for cloned audio.

To generate the voice classification model, 5 machine learning algorithms were used. An ADA model emerged as the most capable of classification, achieving an accuracy of 0.81 (SD 0.05) in 5-fold cross-validation and similar accuracy (0.79) across all testing experiments. The accuracy is in line with previous pause rate detection methods [12], although the testing methodology presented here allows for more comprehensive conclusions about the extendibility of the model results and possible implications for future work. Overall, Model-Naïve participants, a variety of generators, and Model-Naïve paragraphs were used to test the feasibility of the approach.

In the 5-fold cross-validation model optimization, we achieved an accuracy of 0.75 (SD 0.09) for authentic audio and 0.87 (SD 0.08) for cloned audio. Authentic accuracy may have been lower due to the inherent variation in real human speech, as demonstrated by the higher SDs of the pause metrics in Table 1 compared with cloned audio. This could result in decreased performance, as authentic audio may be more likely to overlap with cloned audio features and thus be harder to classify. Furthermore, we did not prioritize authentic speech accuracy in cross-validation, instead optimizing based on balanced class accuracy. Future models could prioritize authentic audio accuracy in model training and hyperparameter tuning if higher authentic accuracy is preferred.

It is important to note that the text the model was tested on had a distinct effect on the performance of the model. In authentic audio samples, the model performed better on known text for both Model-Trained and Model-Naïve participants. Conversely, in Model-Naïve clones, performance improved when the model was tested on a new paragraph. This effect was evident in both pregenerated TTS and Model-Naïve Participant clones for the Model-Trained generators. This may indicate a tendency for the model to slightly overfit to the paragraphs on which it was trained. When exposed to new participants, its performance declines. That being said, the model accuracy for authentic audio from Model-Naïve participants was 0.73. This is within half an SD of the cross-validated authentic audio accuracy (0.75, SD 0.09), further supporting the use of speech pause metrics for robust model prediction.

Incorporating features associated with real, biological processes (such as breathing, thinking, and swallowing) into a deepfake prediction algorithm is likely to enhance its reliability and longevity in the face of ongoing advancements in deepfake technologies. Instead of solely relying on a model trained on the current state of deepfake generation, which may struggle to maintain accuracy as technology evolves, the inclusion of biological features offers valuable insights that enable the model to adapt and effectively detect inauthentic voices. This approach enhances the model’s resilience against evolving deepfake techniques.

### Comparison to Prior Work

High-performance current models are typically trained on spectral or deep-learned audio features obtained from the current state of deepfake generation. This permits for an extremely high accuracy in voice clones in a similar domain to the training data but new advancements and subtle changes in these obscure features could soon make these prediction models obsolete. Indeed, when a high-accuracy prediction model was tested on new, out-of-domain voice clones in a recent study, the prediction accuracy was abysmal (AUC is approximately 25%) [10]. We aimed to evaluate the use of perceptual features in current and future model implementations by testing model performance on a completely new generator. Overall, our model performance on a new generator was a success, and the average accuracy of classification of the new generator was 0.87. This generator provided no audio files for model training, and as such, we can conclude that this technique may be extended to out-of-domain cloning processes.

### Limitations

This research identified certain limitations in the audio quality variation, linguistic diversity, and deepfake generators used in our study. First, since we created a new cloned audio data set, we only had a small amount of data to train and test the prediction model, and the exclusively English-focused experiments did not account for the potential impact of diverse accents or languages on our results. Small data sets may lead to model overfitting, which we attempted to mitigate using a comprehensive model testing methodology. Further exploration in this domain with a larger and more diverse data set encompassing various accents and languages is warranted, as it has the potential to strengthen the robustness of our conclusions and provide a more comprehensive understanding of model performance across linguistic variations.

Second, although the pause rate biomarker enhanced prediction accuracy, it introduced the time requirement of sufficiently long audio samples to accurately calculate pause rate data. An older data set that has been widely used for testing and training previous detection tools consisted of samples shorter than 5 seconds, rendering them incompatible with our model [19]. We prioritize the analysis of longer samples due to their higher potential for misuse in the context of misinformation or impersonation scams. Therefore, our detection tool was optimized for modern voice cloning generators and prioritized longer audio outputs over compatibility with previous deepfake data sets.

Third, another limitation concerns the variation of deepfake generation methods. Our study featured 3 distinct tools to introduce variability in deepfake audio samples. Nevertheless, numerous other models exist and possess subtle distinctions that were not covered in our investigation. While we anticipate that the incorporation of vocal biomarkers will enable accurate predictions regardless of the generation method, we did not test deepfakes produced by alternative tools. This decision stemmed from the recognition that there are numerous methods with slight variations in cloned audio samples, compelling us to focus on some of the most prominent and accessible tools.

### Future Directions

In this study, we aim to show that speech pause metrics may contribute to robust deepfake detection models, and that trained models using these features perform well on out-of-domain data such as new audio deepfake generators or audio samples from new individuals. Further research should perform an ablation study to compare spectral features and pause pattern features, specifically focusing on testing on unknown data.

### Conclusions

In conclusion, the integration of vocal biomarkers into machine learning models shows promise in distinguishing between authentic voice recordings and cloned samples. Given the escalating prevalence of unethical deepfake applications involving impersonation, fraud, and the dissemination of misinformation, establishing a reliable method for verifying source authenticity is crucial. Biological processes and vocal biomarkers offer a potential avenue for enhancing detection methodologies, suggesting a possible means to mitigate the risk of detection tools being rapidly outpaced by advancing deepfake generation technologies.

## Appendix

Multimedia Appendix 1 Speech paragraphs.

Multimedia Appendix 2 Hyperparameter tuning.

## Abbreviations

ADA: AdaBoost
AUC: area under the receiver operator curve
DT: decision tree
LR: logistic regression
RF: random forest
SVM: support vector machine
TTS: text-to-speech
VAD: voice activity detector

## Data Availability

The data sets generated or analyzed during this study are not publicly available due terms in the ethics approval and informed consent. The code used for analysis is available from the corresponding author on reasonable request.
