# Supplementary material for: Investigation of Deepfake Voice Detection Using Speech Pause Patterns: Algorithm Development and Validation
Source: JMIR Biomed Eng. 2024 Mar 21;9:e56245. doi: 10.2196/56245 (PMC11041410; doi:10.2196/56245)
Supplement: Multimedia Appendix 1 [file biomedeng_v9i1e56245_app1.docx]

## Appendix 1 - Speech Paragraphs

**Paragraph 1**

*“The sun beat down on the shimmering sand, as the waves crashed against the shore with a soothing sound. Seagulls screeched overhead, their wings flapping in the breeze. A group of children played nearby, laughing and shouting as they dug holes in the sand and built sandcastles. A distant boat horn echoed through the air, adding to the symphony of sounds. The sound of a passing airplane grew louder and louder, and soon it roared overhead, leaving a trail of white vapor in its wake. The nearby palm trees rustled in the wind, their fronds swaying gently. A nearby beach vendor shouted out his wares, trying to attract the attention of the sunbathers. The smell of sunscreen and salty sea air mingled together, creating a unique aroma that only the beach could produce.”*

**Paragraph 2**

*“The soccer stadium pulsed with an electric energy, reverberating with the heartbeat of the game. Cheers erupted from the passionate crowd as the ball soared through the air, met with thunderous applause and roaring chants. The resounding thud of players' cleats meeting the turf echoed across the field, as they dashed and weaved with nimble grace. The crisp thwack of the ball being struck resounded, accompanied by the collective gasps and anticipatory hush that hung in the air. The referee's whistle pierced the atmosphere, its sharp tone cutting through the noise, signaling a momentary pause in the symphony of sounds. Moments later, the stadium erupted into a crescendo of cheers, as a goal was scored, the net bulging with the weight of triumph. The exultant cries of players filled the air, their shouts of victory mingling with the euphoric jubilation of the crowd. Throughout the match, the rhythmic chorus of synchronized chants rose and fell, creating a pulsating rhythm that fueled the players' determination.”*

**Paragraph 3**

​​*“The bustling city was alive with a cacophony of sounds. Cars honked and screeched as they zoomed by on the busy streets. Pedestrians chatted and laughed as they walked by, their footsteps pounding the pavement. The rhythmic clanging of construction work could be heard in the distance. As I walked past a street performer, the twang of his guitar strings and the mellow notes of his voice filled my ears. Further down the road, a group of children played hopscotch, their cheerful giggles and excited shrieks piercing through the noise. Suddenly, a police siren blared, its sharp wail cutting through the chaos. I covered my ears and hurried away from the commotion, seeking refuge in the relative quiet of a nearby park. The rustling of leaves and the chirping of birds soothed my frayed nerves, reminding me of the simple beauty that exists amidst the noisy bustle of city life.”*
