# Supplementary material for: Investigation of Deepfake Voice Detection Using Speech Pause Patterns: Algorithm Development and Validation
Source: JMIR Biomed Eng. 2024 Mar 21;9:e56245. doi: 10.2196/56245 (PMC11041410; doi:10.2196/56245)
Supplement: Multimedia Appendix 2 [file biomedeng_v9i1e56245_app2.docx]

Appendix 2 – Hyperparameter Tuning

Hypertuned parameters in the classification models.

| Model | LR | ADA | SVM | DT | RF |
| --- | --- | --- | --- | --- | --- |
| Parameter Grid | penalty:  [l1, l2, elasticnet]  C:  [100, 10, 1.0, 0.1, 0.01] | n_estimators:  [10, 50, 100, 500, 750, 1000]  learning_rate:  [0.0001, 0.001, 0.01, 0.1, 1.0] | kernel:  [poly, rbf, sigmoid]  C:  [250, 100, 50, 10, 1.0, 0.1, 0.01] | max_depth:  [2, 10, 15, 20, 50, 100, 150, 200]  min_samples_leaf:  [1, 2, 3, 4, 5] | n_estimators:  [10, 25, 50, 100, 200, 500]  max_depth:  [None, 10, 20, 30, 40, 50] |
| Optimal Parameters | penalty: l2  C: 10 | n_estimators: 500  learning_rate: 0.1 | kernel: rbf  C: 50 | max_depth: 10  min_samples_leaf: 1 | n_estimators: 100  max_depth: None |
